# VCI-Based Analysis on Spatiotemporal Variations of Spring Drought in China

**DOI:** 10.3390/ijerph18157967

**Published:** 2021-07-28

**Authors:** Liang Liang, Siyi Qiu, Juan Yan, Yanyan Shi, Di Geng

**Affiliations:** School of Geography, Geomatics and Planning, Jiangsu Normal University, Xuzhou 221116, China; qiusiyi@jsnu.edu.cn (S.Q.); juanyan@jsnu.edu.cn (J.Y.); syyhealer@jsnu.edu.cn (Y.S.); gengdi@jsnu.edu.cn (D.G.)

**Keywords:** drought, VCI, trend analysis, anomaly index, Mann–Kendall mutation test, wavelet time series analysis

## Abstract

The analysis of spatiotemporal variations in drought is important for environmental monitoring and agricultural production. In this study, the spring vegetative drought conditions in China were analyzed by using the vegetation condition index (VCI) as an indicator to reveal the drought characteristics in China from 1981–2015. The results suggest that spring vegetative drought (especially moderate drought) occurs frequently in China, and drought conditions have obvious geographical differences and are highly affected by monsoons. The frequency of spring vegetative drought is relatively high in the southern and northern regions, which are greatly affected by monsoons, and is relatively low in the northwestern and Qinghai-Tibet regions, which are less affected by monsoons. During 1981–2015, the spring VCI in China showed an overall upward trend. In addition, the trend was not a single change but a wave-like increasing trend that can be divided into four stages: (1) a stage of slow growth from 1981–1990, (2) a stage of intense fluctuations from 1991–2000, (3) a stage of steady growth from 2001–2010, and (4) a stage of slow descent after 2010. The Mann–Kendall test confirmed that the spring VCI in China was increasing, and the changes in the southern, northwestern, and Qinghai-Tibet regions reached significant levels. The time point of mutation in the southern region was 2000, and that in the northwestern and Qinghai-Tibet regions was 1992. Wavelet time series analysis showed that spring vegetation drought in China has a short-period oscillation of 5–7 years and a long-period oscillation of approximately 23–28 years. The northwestern and Qinghai-Tibet regions, which are less affected by the monsoons, are dominated by long-period oscillations, while the southern and northern regions, which are more affected by the monsoons, are dominated by short-period oscillations.

## 1. Introduction

Drought has a serious impact on the environment and agriculture [1]. Drought, especially spring drought, can have a serious impact on crop sowing and growth. Some studies have shown that with the increase in global temperature, the impact of spring drought on wheat, corn and other crops in northern and northwestern China has exceeded the temperature and become the primary factor affecting the yield [2,3,4,5]. Therefore, it is of great significance to monitor spring drought and study its regular occurrence, which can improve the level of agricultural production and promote sustainable development of the national economy.

Drought monitoring indicators can be classified into meteorological observation indicators and remote sensing monitoring indicators. The former includes the standardized precipitation index (SPI), crop moisture index (CMI), Palmer drought severity index (PDSI), etc. These indicators rely on hydrological and meteorological information such as runoff, precipitation, temperature, and soil moisture obtained from ground-based observation sites [6,7,8,9,10,11]. Since the ground observations are based on point data, interpolation calculations are needed to realize spatially continuous monitoring in the application of meteorological observation indicators. Thus, because the number of stations is limited and the distribution is uneven, this approach brings great uncertainty to the drought monitoring results [12,13]. The latter is mainly calculated from remote sensing data, including normalized difference vegetation index (NDVI) [14], temperature vegetation drought index (TVDI) [15,16] and vegetation condition index (VCI) [17], etc. The NDVI is easy to obtain and can be utilized for longer time series than the TVDI. However, it is difficult to obtain ideal results when the NDVI is used to monitor drought in heterogeneous areas due to its sensitivity to the geographical environment [17,18,19]. To overcome these problems, Kogan et al. [17] proposed the VCI index based on the NDVI. The VCI can reflect extreme changes in weather, eliminate spatial changes in the NDVI, reduce the impact of geographical or ecological system variables (mainly weather, soil, vegetation type and terrain), and make different regions comparable.

All these characteristics make the VCI suitable for large-scale drought analysis [17,18,19]. Therefore, the VCI has been widely used in drought monitoring and analysis [19,20,21,22]. For example, Guan et al. [23] analyzed the capabilities of using the VCI for monitoring drought in northwestern China and found that the VCI can commendably reflect the spatial distribution and change in drought. Yan et al. [24] used VCI anomalies as indicators to analyze the development and evolution of drought in five provinces in the middle and lower reaches of the Yangtze River, indicating that the VCI can be used to monitor drought under a wide range of conditions. Ji et al. [25] carried out spring drought monitoring in Qinghai-Tibet by using the VCI, indicating that the index can be used to monitor drought in a large region. The above studies analyzed the situation of vegetation drought in a certain province or region but did not systematically analyze the situation of spring drought in different geographical regions of China. In view of the great impact of spring drought on crops in different regions of China [2,3,4,5], it is of great significance to carry out disaster prevention and mitigation work to analyze the spatiotemporal variation pattern of spring drought in different geographical regions of China by using long-time series spring VCI data.

Therefore, using the long time-series data of spring VCI in China from 1982 to 2015, this paper will solve the following problems: (1) Using frequency statistical method to analyze the occurrence frequency of drought in China; (2) using trend analysis and theMann–Kendall test to study the changing trend and mutation time of spring drought in different geographical regions; and (3) using wavelet analysis to explore the periodic changes and temporal patterns of spring drought in different geographical regions.

## 2. Study Area and Data

### 2.1. Study Area

**Figure 1:**
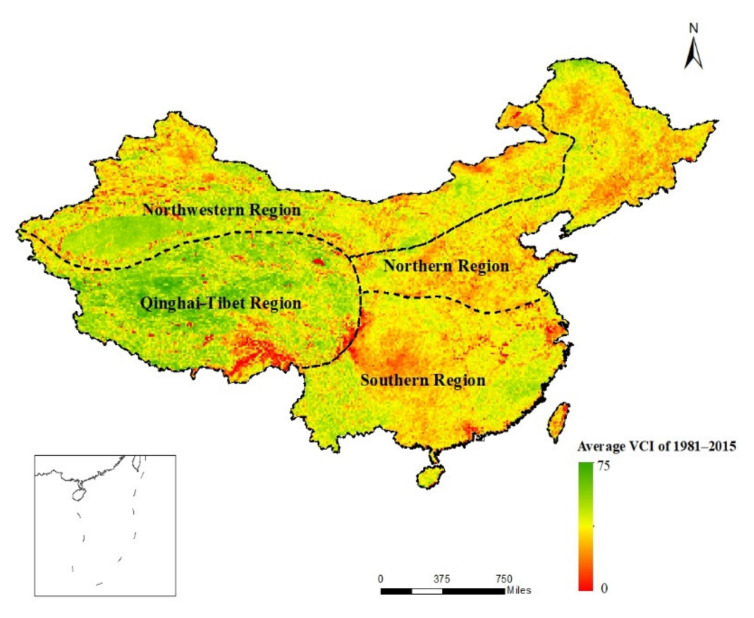
Four geographical regions of China. The background is the average VCI value of 2015.

China’s unique topography and monsoon climate determine the long-term patterns of drought [26]. According to climate, topography, and hydrogeological characteristics, China can be divided into four geographical regions: the northern region, the southern region, the northwestern region and the Qinghai-Tibet region (Figure 1). Among them, the Qinling Mountains–Huaihe River line is the boundary between the Southern and the northern region. The dominant factors are temperature and precipitation, which roughly coincide with the 0 °C isotherm in January and the 800 mm annual isoprecipitation line. The Greater Khingan-Yinshan–Helan Mountains are the boundary between the northwestern region and northern region. The dominant factor is monsoon and non-monsoon, which roughly coincides with the 400 mm annual isoprecipitation line. The Kunlun-Qilian-Hengduan Mountains are the boundary between Qinghai-Tibet region and the other three regions. The dominant factor is topography, that is, the dividing line between the first step and the second step of China [27,28].

### 2.2. Research Data

The VCI data source is the vegetation health product (VHP) dataset provided by NOAA from September 1981 to February 2016, which can be obtained from the official website of NOAA. The spatial resolution of the image data is 4 km, and the temporal resolution is 7 days. There are 52 temporal phases in the whole year. To ensure the integrity of the time series, time interpolation is used to supplement the missing data. According to the definition of Kogan et al. [17], the VCI is a formula that calculates a value between 0 and 100. The larger the VCI value, the better the vegetation growth and the lower the degree of drought [17,18].

In order to study the vegetation drought in spring, it is necessary to calculate the average VCI value in spring by using the above dataset. First, maximum value composites (MVCs) are used to generate the monthly VCI values. This treatment can eliminate some effects of clouds, the atmosphere and the solar altitude angle. Based on the 7-day VCI dataset, this study used MVCs to obtain monthly VCI data on the basis of the following formula:

(1) $$VCI_{i}=Max\left(VCI_{ij}\right),$$

where $VCI_{i}$ is the VCI value in month *i* and $VCI_{ij}$ is the VCI value of week *j* in month *i*. Based on the seasonal division of meteorology on the solar calendar, spring runs from March to May on the solar calendar. Therefore, the spring average VCI is defined as follows:

(2) $$\bar{VCI_{S}}=\left(\overset{5}{\underset{i=3}{\sum }}VCI_{i}\right)/3,\;i=3,\;4\;,5\;\;,\;$$

where $\bar{VCI_{s}}$ is the cumulative average VCI in March, April and May, $VCI_{i}$ is the maximum value of the VCI in month *i*, and *i* is the number of months.

Based on the above method, the spring VCI time-series data from 1982 to 2015 are obtained.

## 3. Research Methods

### 3.1. Calculation of Drought Frequency

To describe the dynamic spatiotemporal variations in spring vegetative drought, the VCI values were divided into four grades according to the drought classification criteria: no drought (*VCI* > 70), slight drought (50 < *VCI* ≤ 70), moderate (30 ≤ *VCI* ≤ 50) and severe (*VCI* < 30) [29,30]. The drought frequency was then calculated by the following formula:

(3) $$f=\frac{n}{N}\times 100\%$$

where *f* is the frequency of drought, *n* is the number of drought occurrences during the research period, and *N* is the total length of the research period.

### 3.2. VCI Trend Analysis

To study the overall trends of changes in the research period, the trends were calculated according to the VCI values of each pixel over 35 years. The specific formula is as follows [31]:

(4) $$Slope=\frac{n\times \sum_{i=1}^{n}x_{i}t_{i}-\sum_{i=1}^{n}x_{i}\sum_{i=1}^{n}t_{i}}{n\times \sum_{i=1}^{n}t_{i}^{2}-{\left(\sum_{i=1}^{n}t_{i}\right)}^{2}}$$

where $t_{i}$ is the serial number from 1981 to 2015 (1–35), *n* is the total length of the time series (*n* = 35), and $x_{i}$ is the VCI value in year *i*. In addition, *slope* > 0 indicated that the VCI value showed an upward trend, and the drought degree increased with increasing time during the research period; otherwise, it showed a downward trend.

To avoid autocorrelation in the VCI sequence affecting the results of trend analysis, before the trend analysis, autocorrelation tests on VCI mean series of different geographical regions in spring were carried out. The *F*-test was used to determine the significance of changes. The data follows the F distribution, and the degrees of freedom are (1, *n*−2), where *n* represents 35 years. According to the F distribution table, $F_{0.05(1,33)}=4.14$ and $F_{0.01(1,33)}=7.56$. According to these thresholds and the slope value, the trend of the VCI can be divided into six grades (Table 1). The above operations were completed in SPSS 18.0 and ArcGIS.

### 3.3. Calculation of the Anomaly VCI

The anomaly vegetation condition index (AVCI) is used to analyze historical variations in the VCI. The definition of the AVCI is as follows [24]:

(5) $$AVCI=(VCI_{i}-VCI_{\mathrm{ave}})∕VCI_{\mathrm{ave}}$$

where *AVCI* is the abnormal VCI, $VCI_{i}$ is the VCI value in a specific time period, and $VCI_{ave}$ is the average VCI over the research period. A positive AVCI indicates rich soil moisture and better vegetation conditions, and a negative AVCI indicates that the soil moisture is relatively deficient and the vegetation conditions are worse than average.

### 3.4. Mutation Test for the VCI

To determine whether there was a mutation during the research period, the Mann–Kendall mutation test is usually performed on the VCI dataset. The Mann–Kendall test is a nonparametric statistical test [32] with the assumption of an independent random time series and is defined as follows:

(6) $$UF_{k}=\frac{\left[s_{k}-E(s_{k})\right]}{\sqrt{Var(s_{k})}},(k=1,2,\ldots ,n)$$

where $s_{k}=\sum_{i=1}^{k}r_{i}$ and $r_{i}$ is the number of samples that are between 1 and i and are less than $x_{i}$. Under the zero assumption, the mean and variance are, respectively:

(7) $$E(s_{k})=\frac{n(n-1)}{4}$$

(8) $$Var(s_{k})=\frac{n(n-1)(2n+5)}{72}$$

This process was repeated for the inverse time series to calculate $UB_{k}$, and then the curves of $UF_{k}$ and $UB_{k}$ were plotted in Excel by using a confidence interval of 0.05 ($U_{0.05}=\pm 1.96$). The Mann–Kendall test can effectively distinguish whether a natural process is in a state of natural random fluctuation or has a certain trend. $UF_{k}$ or $UB_{k}$ values greater than 0 indicate an increasing trend; in contrast, $UF_{k}$ or $UB_{k}$ values smaller than 0 indicate a decreasing trend. If the confidence interval of $UF_{k}$ or $UB_{k}$ exceeds 0.05, it indicates that there is a significant trend, and the range beyond the confidence interval is regarded as the mutation period; if there is an intersection between $UF_{k}$ and $UB_{k}$ and it is in the confidence interval, the time corresponding to the intersection is regarded as the beginning of mutation [30,32].

### 3.5. Wavelet Analysis of the VCI

Wavelet analysis, known as a “mathematical microscope”, can automatically adapt to the requirements of time–frequency signal analysis; therefore, it focuses on any particulars of the signal arbitrarily and is widely used in signal processing [33]. For time series analysis, due to the characteristics of multiresolution analysis, the wavelet transform can reflect the local characteristics of the signal in both time and frequency domains. Therefore, the wavelet transform can obtain the wave pattern of a time series (including the temporal pattern of periodic changes) by decomposing the time series into the time-frequency domain [34]. Among all kinds of wavelets, Morlet wavelets can balance the localization of time and frequency and are widely used in noise elimination, feature signal extraction, image processing and other fields [35]. Therefore, the Morlet wavelet is used in this paper to analyze the VCI index of China and its four geographical regions from 1981 to 2015 and to explore the periodic changes and temporal patterns of vegetation drought in each region [36]. The continuous Morlet wavelet is defined as:

(9) $$\varphi \left(t\right)=\pi^{-1/4}\exp\left(-iw_{0}t\right)\exp\left(-t^{2}∕2\right),$$

where the Morlet wavelet $\varphi \left(t\right)$ is the result of sine complex $\exp\left(-iw_{0}t\right)$ and Gaussian wave package $exp\left(-t^{2fon}/2\right)$, *t* is the dimensionless time, $w_{0}$ is the dimensionless frequency, and $\pi^{-1/4}$ is a standardized factor to ensure unit variance [37].

## 4. Results and Analysis

### 4.1. Drought Frequencies in China

**Figure 2:**
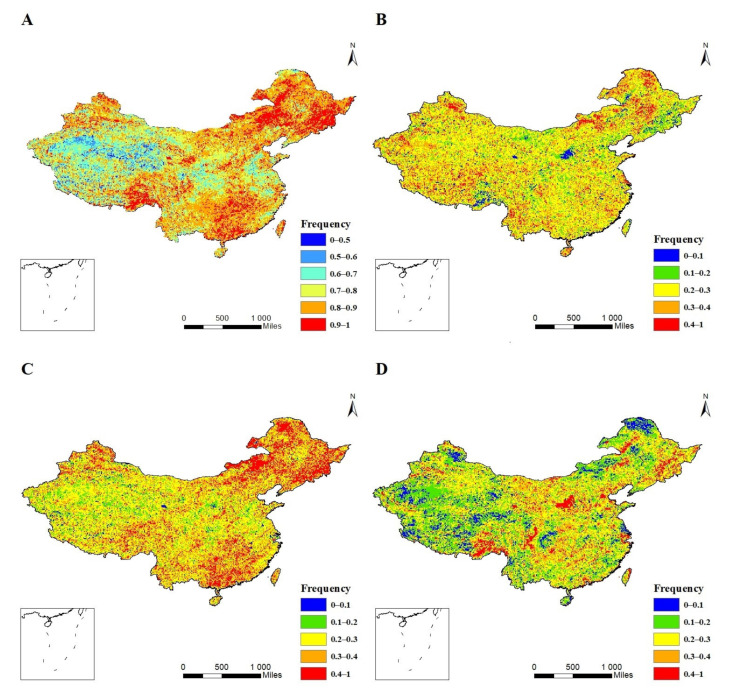
Spatial distribution of drought occurrence frequencies for different drought grades during spring in China. (**A**) total drought occurrence frequencies; (**B**) slight drought occurrence frequencies; (**C**) moderate drought occurrence frequencies; (**D**) severe drought occurrence frequencies.

According to the classification of VCI drought grades in Table 1, the spatial distribution of vegetation drought frequency in spring from 1981 to 2015 in China was calculated by Formula (2). Spring drought occurred frequently and exhibited apparent regional differences from 1981 to 2015 (Figure 2A). The most serious spring drought was in the northern region, where the frequency of drought was above 0.8 and drought occurred in 67.11% of the region; the second most serious spring drought was in the southern region, where most parts of Jiangxi, Hunan, Guangxi and Guangdong had a drought frequency greater than 0.8. In the northwestern and Qinghai-Tibet regions, the frequency of drought was relatively low; except for parts of southwestern Tibet, the frequency of drought in most areas of the Qinghai-Tibet Plateau was less than 0.7. Generally, the regions affected by monsoons in China, i.e., the northern and southern regions had a high incidence of spring vegetation drought because of the large interannual hydrothermal changes; the frequency of spring vegetation drought was relatively low in northwestern China and the Qinghai-Tibet region, and drought was serious in only a small area of northern Inner Mongolia, northern Xinjiang and southern Tibet.

To further study the vegetation drought intensity in China, the frequency of spring drought occurrence in different grades was analyzed (Figure 2B–D). The results showed that although the frequency of spring drought in China was generally high, the severe drought frequency was relatively low, and most of the drought frequencies were slight or moderate (especially moderate drought). Slight spring drought occurs all over the country. Moderate spring droughts occur more frequently in northern and southern China. In the northern region, moderate spring drought mainly occurs in Heilongjiang, Jilin and Liaoning provinces and the northern part of the Inner Mongolia Autonomous Region, while in the southern region, moderate spring drought mainly occurs in Hunan, Guangdong and Guangxi, as well as western Guizhou and eastern Jiangxi.

### 4.2. Analysis of Drought Trend in China

**Figure 3:**
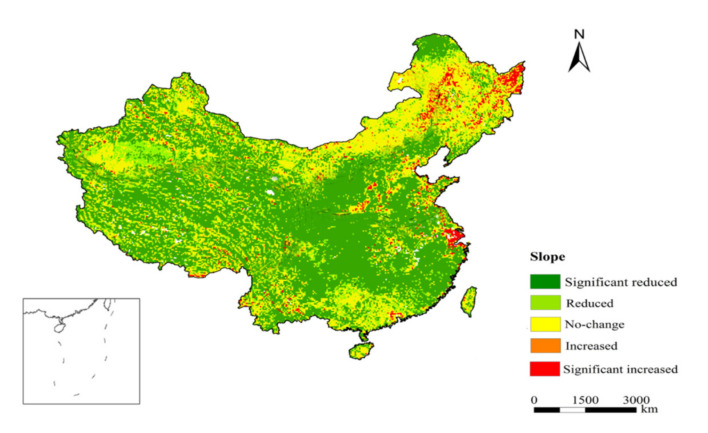
Slope trend of the average VCI in spring from 1981–2015 in China.

The trend analysis method combined with the F-test was used to analyze the trends of the VCI in spring from 1981 to 2015 in China (Figure 3), and the different trend levels of the VCI were counted. The VCI showed the most significant growth trend in spring, and the VCI increased in 64.74% of the pixels and significantly increased in 53.2% of the land area. The VCI decreased in only a small part of the region, approximately 4.4%, and the VCI decreased significantly in 2.94% of the areas, mainly in the eastern part of Heilongjiang, the eastern part of Inner Mongolia and some coastal areas of Jiangsu and Zhejiang provinces. In addition, the VCI in the southeastern coastal area and the Yangtze River Delta area decreased significantly in the three seasons, and the trend was obviously different from that in the surrounding areas. This is probably because of rapid urbanization instead of drought. This is also a limitation of using the VCI to analyze drought; that is, some drastic changes in vegetation caused by artificial changes can be mistaken for drought changes. Therefore, whether the change in vegetation drought is driven by human factors or natural factors is a question for further study.

### 4.3. Analysis of Temporal Drought Characteristics in Different Geographical Regions

**Figure 4:**
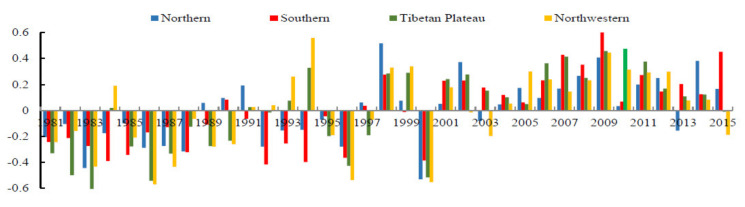
Interannual changes in spring AVCI in different geographic areas.

To analyze the temporal characteristics of drought in different parts of China, the AVCI during different seasons in different regions from 1981 to 2015 was analyzed (Figure 4). From 1981 to 2015, the spring AVCI increased slowly, and the trends of the four geographical regions were consistent, indicating that the spring drought eased gradually, which showed no difference from the analysis above. Specifically, the period from 1981–2015 can be divided into four stages. From 1981 to 1990, except for the shock caused by the super-strong El Niño phenomenon in approximately 1983, most of the AVCI values in all parts of the country were less than the mean value, but the overall trend of AVCI was increasing, indicating that in the 1980s, China was in drought, but the drought was gradually alleviated. From 1991 to 2000, in general, was a transitional period of fluctuating AVCI values, which reached their minimum in 2000. However, this process was influenced by the super El Niño phenomenon in approximately 1988, so the AVCI oscillated violently during this time, including during the abundant rainfall and frequent floods around the country in 1998 and the severe droughts in 2000. This was a period of large interannual changes in drought during the Spring Festival in China, with frequent floods and droughts. From 2001–2010, the AVCI in each region increased, marking another period of growth, and reached peak values in 2010. In 2010–2015, the AVCI decreased. It can be inferred from the above analysis that the decreasing AVCI phase will continue for some time in the future. Although the drought trend is decreasing in China, drought is also currently in a small decreasing cycle. Notably, after the national drought in 2000, the AVCI was above the average value and reached its maximum value in 2009. This change may be due to the return of farmland to forest in 1999, which lasted for several years.

### 4.4. Mutation Analysis of VCI Time Series in Different Geographical Regions

**Figure 5:**
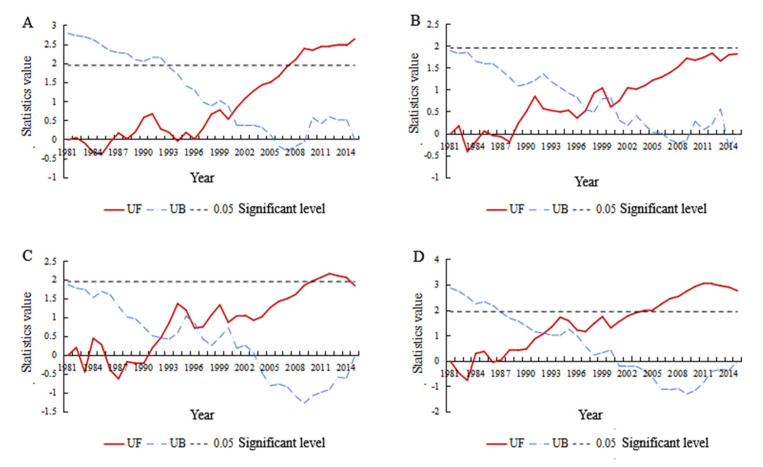
Mann–Kendall mutation analysis results of VCI time series for the southern (**A**), northern (**B**), northwestern (**C**) and Qinghai-Tibet (**D**) regions of China.

The Mann–Kendall test was used to determine if the temporal variations in the VCI from 1981 to 2015 were random fluctuations or obvious trends. According to the definition of Mann–Kendall, the UF and UB statistics of the VCI time series in the same season were calculated. The UF and UB curves and the 0.05 significance level (U0.05 = 1.96) were plotted in Excel. The results are shown in Figure 5.

According to the figure, most UF curves of the four geographical regions in China were greater than zero, which indicates that the seasonal VCI increased over time. This result is consistent with the statistical analysis results given in Section 3.2; that is, the area with increasing VCI in spring was relatively large (approximately 64.74% of the area had increasing VCI). The UF and UB curves intersected in the confidence interval in 2000 and passed the significance test at the 0.05 level in 2007 (U0.05 = 1.96), which showed that there was a mutation in the VCI increase in spring in the southern region of China and that the mutation point was 2000 (Figure 5A). The UF and UB curves of the spring VCI in the northern region of China had three intersections in the confidence interval, which were located in 1997, 1999 and 2001. However, the upward trend of the UF curves did not exceed the 0.05 significance level; that is, the change did not reach a significant level (Figure 5B). The UF and UB curves of spring VCI sequences in the northwestern region of China had three intersections in the confidence interval; the points intersected in 1992 and 1996, and the UF curves exceeded the 0.05 significance level in 2010. To determine the specific year in which the mutation occurred, the cumulative anomaly method was used to analyze the mutation (Figure 5C). It was found that after reaching the lowest point in 1990, although there were fluctuations in the following years, the overall trend was upward, indicating that the mutation occurred in 1992; the UF and UB curves of spring VCI in the Qinghai-Tibet region intersected in the confidence interval in 1992, and exceeded 0.05 significance level in 2004, indicating that the upward trend of spring VCI in Qinghai-Tibet region was significant, and as in the northwestern region, the mutation occurred in 1992 (Figure 5D).

### 4.5. Wavelet Analysis of the VCI in Different Geographical Regions

**Figure 6:**
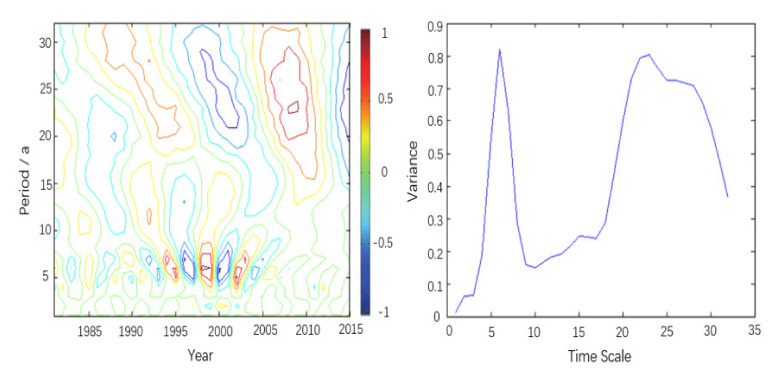
Wavelet time series analysis of spring VCI in China, 1981–2015.

On the annual scale from 1981 to 2015, the Morlet continuous complex wavelet transform, which is commonly used in hydrometeorology, was used to analyze the spring VCI values in China and its four geographical regions [38]. The wavelet coefficient contour map can reflect periodic changes in the VCI and its distribution in the time domain at different time scales to judge the periodic variability of dry and wet conditions in China at different time scales in the future. The wavelet variance graph can reflect the distribution of the wave energy over different scales in the time series and can be used to determine the major period in the process of change. The contour map and wavelet variance diagram of the real part of the wavelet coefficients in Figure 6 shows that there are obviously short periods of 4–8 years and 10–16 years and long periods of 19–32 years on the annual scale. Among them, there were seven obvious alternations of drought and humidity in the time scale of 4–8 years, four obvious alternations of drought and humidity in the time scale of 10–16 years, and one obvious alternation of drought and humidity in the time scale of 19–32 years. Combined with the wavelet variance graph, it can be seen that there are obvious oscillations on the time scales of 6, 23 and 27 years, and 23 and 6 years are the main periods. The wavelet coefficient graph of 2015 is not closed in the 23-year long period or the 6-year short period, which shows that it continued to be wet in the 6-year short period and dry in the 23-year long period.

**Figure 7:**
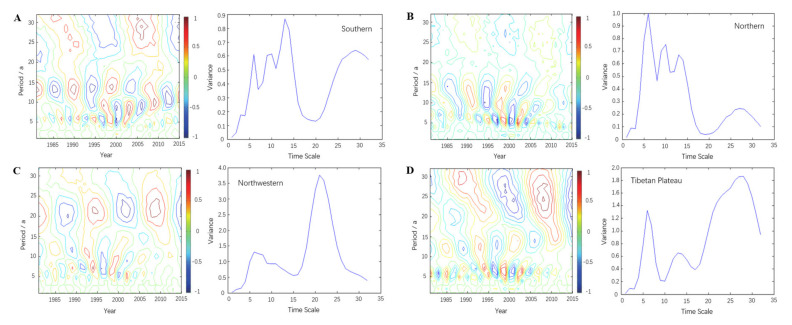
Spring VCI wavelet time series analysis maps for the southern (**A**), northern (**B**), northwestern (**C**) and Qinghai-Tibet (**D**) regions of China.

To further analyze the change in vegetation drought in different geographical regions, the VCI in spring in the southern, northern, northwestern and Qinghai-Tibet regions of China was analyzed. There are 6-, 10-, 14- and 29-year oscillation periods in the spring VCI in southern China. Among them, the signal in the 14-year cycle is the strongest, which is the first main cycle, and the second and third cycles are 6 years and 10 years, respectively (Figure 7A). On a scale of 14 years and 10 years, spring in this region was in a period of partial humidity in 2015, and there was a trend of continuous humidity. However, on the six-year scale, southern China was in a drought-prone period in spring, and there was a trend of continuous drought proneness after 2015. As shown in Figure 7B, in the spring of 1981–2015, the VCI in the northern region showed obvious alternations of high and low values on the scales of 4–8 years, 9–18 years and 21–32 years. The wavelet variance shows that 6 years, 10 years and 13 years are the first, second and third main periods, respectively. In the six-year main cycle, the VCI in the northern region experienced eight cycles of alternating vegetative drought and humidity from 1981 to 2015. The year 2015 was in the transitional period of alternating drought and humidity, and after that, there was a tendency of partial humidity in spring in northern China. According to the wavelet coefficient and wavelet variance map of spring VCI in northwestern China, the first main period is at 21 years and the second and third periods are at 6 and 11 years, respectively (Figure 7C). On a scale of 21 and 11 years, 2015 was in a period of drought, and on the scale of 6 years, it was in a period of humidity. The spring VCI in the Qinghai-Tibet region has obvious oscillations in the 6-year, 13-year and 27-year periods (Figure 7D). Among them, the energy of the 27-year scale is the strongest, which is the first main period, and the second and third periods are 6 years and 13 years, respectively. In the period of 27 years and 13 years, the Qinghai-Tibet region is in a period of partial drought and has a trend of continuous partial drought; however, on the six-year scale, the regional spring is currently in a period of partial humidity, and there is a trend of continuous partial humidity after 2015. That is, the region is in a small humid period within a large drought-prone period. Comprehensive analysis of the four geographical regions shows that in the northwestern and Qinghai-Tibet regions of China, which are less affected by the monsoon, oscillations are more obvious in the long period, while in southern and northern China, which are more affected by the monsoon, oscillations are more obvious in short periods.

## 5. Discussion

Spring drought is one of the main natural disasters and has a serious impact on the social economy [39]. Through the assessment of drought conditions over time, we can provide a reference that is the basis for the formulation and implementation of early warning and measures of drought resistance. Based on the drought frequency, trend, Mann–Kendall test and wavelet time series analysis, the VCI is taken as the drought index in this paper to analyze the spatial and temporal variations in spring drought in China at a macroscale. The analysis shows that China is a country with frequent spring drought, but mainly moderate drought and light drought, and drought has tended to ease on the whole. This conclusion is consistent with results based on the PDSI to analyze the temporal and spatial evolution of drought in China but slightly different from a study based on the SPI [40,41,42]. This is because the SPI reflects the situation of meteorological drought, while the VCI represents the actual effect of meteorological drought on vegetation, reflecting the comprehensive influence of precipitation, temperature, evapotranspiration and other factors. The PDSI is similar to the VCI, which integrates the effects of temperature, humidity, evaporation and recharge rate. Therefore, compared with the normalized precipitation index, the VCI can better reflect the actual impact of meteorological drought on vegetation.

Wavelet analysis can be used to study trends, periodicity, randomness, mutations and “multi time scale” structures in time series and can clearly reveal various changes hidden in nonstationary time series, such as those of precipitation, temperature and humidity [43,44]. In this paper, Morlet wavelet analysis is used to analyze the variation in vegetation drought in four geographical regions of China. The results show that there are short-period oscillations of approximately 5–7 years and long-period oscillations of approximately 23–28 years in different regions of China. Among them, the northwestern and Qinghai-Tibet regions, which are less affected by the monsoon, are dominated by long-period oscillations with relatively small amplitudes; the southern and northern regions, which are more affected by the monsoon, are dominated by short-term oscillations with relatively large amplitudes. This further indicates that the drought frequency is higher in the northern and southern regions of China, and lower in the northwestern and Qinghai-Tibet regions. Due to the influence of the spring monsoon in the southern and northern regions, the water and heat conditions change sharply in a short period, resulting in frequent drought in the regions. In contrast, the influence of the spring monsoon in the northwestern and Qinghai-Tibet regions is lower, and the water and heat conditions change slowly over a long period, so the spring drought frequency is relatively low. The results of this paper are similar to those of Qian [21] and Shen [22] in the spatial distribution of vegetation drought, i.e., the frequency and trend of spring drought in China have obvious spatial differences, and compared with the northwestern and Qinghai-Tibet regions, the southern and northern regions affected by monsoons have more frequent spring drought.

Different from the NDVI and other indices that directly reflect the vegetation growth status, the VCI reflects the difference between the vegetation status and the background value in a region, so it can characterize the drought situation according to the local climate and natural background conditions. Therefore, a series of studies have shown that using the VCI for drought analysis can reduce or eliminate the influence of geographical, climatic and soil factors [17,18,19,20,21,22]. This study also confirms this point. The results show that the spring vegetation drought frequency in the southern region is higher than that in the northwestern and Qinghai-Tibet regions. This is because although the northwestern region is traditionally an arid region, according to the theory of evolution, the local vegetation and crops that have grown for a long time in an area are adapted to the local climate, soil and other natural conditions. Therefore, as long as there is no abnormal situation in the area, the vegetation can grow normally, the local ecological environment will not be damaged, and agricultural production can be guaranteed. The northwestern and Qinghai-Tibet regions are less affected by the monsoon, and the climate is relatively stable, so the frequency of spring drought is low, while the southern and northern regions are affected by the monsoon, and the rainfall and temperature change greatly, so spring drought occurs frequently. Therefore, the VCI is a good indicator of drought and can objectively reflect the drought in a region, whether it is humid and rainy in the southern region or arid and rainless in the northwestern region.

In addition, although this study focuses on the analysis of spring drought in China, considering that VCI can reduce the impact of geographical or ecological system variables and make different regions comparable, the methods can also provide a reference for drought monitoring in other places. In the next step, VCI can be used to monitor global drought and a method such as ARIMA (Autoregressive Integrated Moving Average model) can be used to build a time-series prediction model to provide drought early warning for the world.

## 6. Conclusions

Drought has caused tremendous damage to human survival and economic development in different countries [1]. It is of great significance to analyze the spatial and temporal variations in drought for the formulation and implementation of drought resistance and disaster reduction policies. In this study, the NOAA/AVHRR dataset was used to acquire VCI data for China from 1981 to 2015, and the spatial and temporal characteristics of drought over the past 35 years were analyzed. The major findings include the following:

1. China has a high frequency of spring drought but mainly suffers from slight and moderate drought. Moreover, there are obvious regional differences in spring drought. The frequency of spring drought is higher in the southern and northern regions, which are more affected by the monsoon; except for northern Xinjiang and southern Tibet, the frequency of drought is relatively low in the northwestern and Qinghai-Tibet regions, which are less affected by the monsoon.
2. During 1981–2015, the spring VCI in all parts of China showed an overall upward trend; that is, the spring drought in most regions tended to ease. Temporal analysis showed that the trend was not a single change but a wavy growth trend, which can be divided into the stage of slow growth from 1981–1990, the stage of sharp fluctuations from 1991–2000, the stage of steady growth from 2001–2010 and the stage of slow decline after 2010.
3. The Mann–Kendall test further indicated that since the 1990s, the change in the VCI series was an actual upward trend instead of a random fluctuation, and the changes in the southern, northwestern and Qinghai-Tibet regions reached a significant level. Among them, the time point of mutation in the southern region was 2000, and in the northwestern and Qinghai-Tibet regions, it was 1992.
4. There are short-period oscillations of approximately 5–7 years and long-period oscillations of approximately 23–28 years in China and its four geographical regions. Among them, the northwestern and Qinghai-Tibet regions, which are less affected by the monsoon, are dominated by long-term oscillations, while the southern and northern areas, which are more affected by the monsoon, are dominated by short-term oscillations. All regions have approximately seven obvious alternations of drought and humidity on the short-cycle scale and two obvious alternations of drought and humidity on the long-cycle scale. In addition, all regions are in a small humid period within a large partial drought cycle.

## Figures and Tables

**Table 1:** Different change grades of VCI defined by trend analysis and F test.

Slope of Average VCI	*F*-Test Value	Change Level of VCI
*slope* > 0	F ≥ 7.56	Extremely significant increase
	4.14 ≤ F < 7.56	Significant increase
	F < 4.14	No significant increase
*slope* < 0	F < 4.14	No significant decrease
	4.14 ≤ F < 7.56	Significant decrease
	F ≥ 7.56	Extremely significant decrease

## Data Availability

Data may be provided on reasonable request to corresponding author.

## References

[1] Gu Y., Ni S.H., Lin J. (2011). China’s drought disaster situation changes and distribution characteristics. China Water Resour..

[2] Ma S.Q., Wang Q., Yu H., Xu L.P., Zhang T.L. (2012). Field experiment study into influence of spring drought on maize yield. J. Nat. Disasters.

[3] Chen S.Y., He H.M., Wu F.R., Li C.D. (2015). Climate change characteristics of the spring drought in southwest-south China. J. Arid Land Resour. Environ..

[4] Cao Y.Q., Zheng S., Fan S.B., Guo M. (2017). Evolution analysis of spring drought events in Northwest Liaoning based on grade adjustment of Z-Index. Arid Land Geogr..

[5] Yu F.Y., Jin L.Y., Li J.J. (2020). Characteristics of Spring Drought in Southwest China and the Anomalous Circulation. Desert Oasis Meteorol..

[6] Raziei T., Bordi I., Pereira L.S. (2013). Regional drought Modes in Iran using the SPI: The effect of Time scale and spatial resolution. Water Resour. Manag..

[7] Hao Y., Wang S.Q., Wang J.B., Lu H.Q., Guo A.H., Zhu Z.C. (2016). Assessing spatiotemporal variation of drought in China and its impact on agriculture during 1982–2011 by using PDSI indices and agriculture drought survey data. J. Geophys. Res..

[8] Wei J., Ma G.Z. (2003). Comparison of Palmer Drought Severity Index, percentage of precipitation anomaly and surface humid index. Acta Geogr. Sin..

[9] Feng Q., Tian G.L., Wang A.S., McVicar T.R., Jupp D.L.B. (2004). Remote sensing monitoring of soil humidity using vegetation condition index. J. Nat. Disasters.

[10] Li Z., Zhou T., Zhao X. (2016). Diverse spatial-temporal responses in vegetation growth to droughts in China. Environ. Earth Sci..

[11] Guo N., Guan X.D. (2007). An improvement of the vegetation condition index with applications to the drought monitoring in Northwest China. Adv. Earth Sci..

[12] Mirabbasi R., Anagnostou E.N., Fakheri-Fard A., Dinpashoh Y., Eslamian S. (2013). Analysis of meteorological drought in northwest Iran using the joint deficit index. J. Hydrol..

[13] Mendicino G., Senatore A., Versace P. (2008). A groundwater resources index (GRI) for drought monitoring and forecasting in a Mediterranean climate. J. Hydrol..

[14] Rouse J., Haas R.H., Schell J.A. (1974). Monitoring vegetation systems in the Great Plains with ERTS. NASA Spec. Publ..

[15] Liang L., Zhao S., Qin Z., He K., Chen C., Luo Y. (2014). Drought change trend using MODIS TVDI and its relationship with climate factors in China from 2001 to 2010. J. Integr. Agric..

[16] Sandholt I., Rasmussen K., Andersen J. (2001). A simple interpretation of the surface temperature/vegetation index space for assessment of surface moisture status. Remote Sens. Environ..

[17] Kogan F.N. (1990). Remote sensing of weather impacts on vegetation in non-homogeneous areas. Int. J. Remote Sens..

[18] Kogan F.N. (1995). Droughts of the late 1980s in the United States as derived from NOAA polar-orbiting satellite data. Bull. Amer. Meteor. Soc..

[19] Liu W.T., Kogan F.N. (1996). Monitoring regional drought using the Vegetation Condition Index. Int. J. Remote Sens..

[20] Deng M., Di L., Han W., Yagci A., Peng C., Heo G. (2013). Web-service-based Monitoring and Analysis of Global Agricultural Drought. Photogramm. Eng. Remote Sens. PERS.

[21] Qian X., Liang L., Shen Q., Sun Q., Zhang L., Liu Z., Zhao S., Qin Z. (2016). Drought trends based on the VCI and its correlation with climate factors in the agricultural areas of China from 1982 to 2010. Environ. Monit. Assess..

[22] Shen Q., Liang L., Luo X., Li Y., Zhang L. (2017). Analysis of the spatial-temporal variation characteristics of vegetative drought and its relationship with meteorological factors in China from 1982 to 2010. Environ. Monit. Assess..

[23] Guan X.D., Guo N., Huang J.P., Ge J.P., Zheng Z.H. (2008). Applicability analysis of VCI to monitoring northwest China drought. Plateau Meteorol..

[24] Yan Y., Xiao F., Du Y., Ling F., Li X.D., Li Y.Z. (2012). Monitoring droughts in the five provinces along the middle-lower reaches of the Yangtze River during spring/summer 2011 using AVCI. Resour. Environ. Yangtze Basin.

[25] Ji M., Zhang C., Zhao J.W., Yan J., Liang L. (2021). Temporal and spatial dynamics of spring drought in Qinghai-Tibet region based on VCI index. Remote Sens. Land Resour..

[26] Feng Q., Tian G.L., Liu Q.H. (2003). Research on the operational system of drought monitoring by remote sensing in China. J. Remote Sens..

[27] Han Y.F. (2000). China Regional Geography.

[28] Zhao J., Chen C.K. (2011). China’s Geography.

[29] Chen L., Ford T.W., Yadav P. (2021). The Role of Vegetation in Flash Drought Occurrence: A Sensitivity Study Using Community Earth System Model, Version 2. J. Hydrometeorol..

[30] Liang L., Sun Q., Luo X., Wang J., Zhang L., Deng M., Di L., Liu Z. (2017). Long-term spatial and temporal variations of vegetative drought based on vegetation condition index in China. Ecosphere.

[31] Zhang Y.D., Zhang X.H., Liu S.R. (2011). Correlation analysis on normalized difference vegetation index (NDVI) of different vegetations and climatic factors in Southwest China. Chin. J. Appl. Ecol..

[32] Wei F.Y. (1999). Statistical Diagnosis and Prediction Technology of Modern Climate.

[33] Liu M.C. (2013). Wavelet Analysis and Its Application.

[34] Torrence C., Compo G.P. (1998). A practical guide to wavelet analysis. Bull. Am. Meteorol. Soc..

[35] Liu Y., Xu G., Yin Z., Hu C., Wang Y., Liao F. (2017). Spatio-temporal change of surface air temperature in Anhui province in the context of global warming from 1960 to 2014. J. Nat. Resour..

[36] Li M., Wang G.W., Chai X.R., Hu W.X., Zhang L.Z. (2019). Spatial clustering based climatic zoning and drought time variation characteristics in northeast China. J. Nat. Resour..

[37] Lara C., Saldías G.S., Paredes A.L., Cazelles B., Broitman B.R. (2018). Temporal Variability of MODIS Phenological Indices in the Temperate Rainforest of Northern Patagonia. Remote Sens..

[38] Zhang Q., Tang H.P., Cui F.Q., Dai L.W. (2019). Based on the variation characteristics of the hulun buir grassland drought SPEI and trend analysis. Acta Ecol. Sin..

[39] Dai A. (2011). Characteristics and trends in various forms of the Palmer Drought Severity Index during 1900–2008. J. Geophys. Res..

[40] Suping W., Cunjie Z., Yaohui L., Jianying F., Jinsong W. (2014). Analysis of mult-timescale drought variation based on standardized precipitation index in China during 1960–2011. J. Desert Res..

[41] Wang Z., Li J., Huang Z., Zhong R., Chen J., Qiu Z. (2016). Spatiotemporal variations analysis of meteorological drought in China based on scPDSI. Trans. Chin. Soc. Agric. Eng..

[42] Zhang J., Shen Y.J. (2019). Spatio-temporal variations in extreme drought in China during 1961–2015. J. Geogr. Sci..

[43] Polanco-Martínez M., Fernández-Macho J., Medina-Elizalde M. (2020). Dynamic wavelet correlation analysis for multivariate climate time series. Sci. Rep..

[44] Anshuka A., Buzacott A.J., Vervoort R.W., van Ogtrop F.F. (2021). Developing Drought Index Based Forecasts for Tropical Climates using Wavelet Neural Network: An Application in Fiji. Theor. Appl. Climatol..

